# Beyond the global attachment model: domain- and relationship-specific attachment models at work and their functions

**DOI:** 10.3389/fpsyg.2023.1158992

**Published:** 2023-05-18

**Authors:** Katarína Greškovičová, Elena Lisá

**Affiliations:** Faculty of Social and Economic Sciences, Institute of Applied Psychology, Comenius University in Bratislava, Bratislava, Slovakia

**Keywords:** adult attachment, attachment at work, attachment working models, attachment types, attachment orientations, cluster analysis, attachment hierarchy, workplace attachment

## Abstract

**Introduction:**

Since prior research has shown the importance of specific attachment models, we wanted to explore specific adult attachments (colleagues, leader, and workplace) in the working setting. The study aimed to investigate the position of specific adult attachments in the attachment hierarchy and their associations with various organizational variables. Finally, we assumed that dimensions of the colleagues-domain attachment model would cluster into attachment types at work, according to secure, preoccupied, and avoidant attachment orientations.

**Methods:**

We carried out cross-sectional time-lagged research design. The sample consisted of 1,352 participants based on convenience and voluntary sampling procedures. Participants aged 18–78 worked in various work teams and positions. The battery consisted of the Adult Attachment in the Workplace Questionnaire, the Workplace Attachment Styles Questionnaire, the Scale of Belonging to the Organization, the Leader as Security Provider Scale, the shortened Experiences in Close Relationships-Revised Questionnaire, the Czech Leadership Questionnaire, the Citizenship Organizational Behavior Questionnaire, the General Work Performance questionnaire, the Utrecht Work Engagement Scale. Data were analyzed in JASP 0.16.3 and IBM SPSS Statistics 22. Among other statistical methods, we performed factor analysis and two-step cluster analysis. The alpha level for statistical testing was set to 0.05.

**Results:**

The results show that the work-specific attachment models differ from the romantic domain attachment model. Moreover, the work-specific attachment models also differ among themselves. Depending on the attachment to colleagues, it is possible to distinguish two attachment orientations (secure and insecure) among working adults. These two types differ in all the variables studied (relationships with colleagues, romantic partners, belonging to people and place, and performance).

**Conclusion:**

The study advances our knowledge of attachment working models and their application in the organizational context. We confirmed hierarchical attachment mental presentations and show the distinction in attachment working models at work. Colleagues and leaders form two separate domains within the workplace. Attachment to a leader is associated with the leadership style and secure workplace attachment. Attachment to colleagues might be more important in insecure workplace attachment and insecure belonging. Fostering secure attachment at work might bring together more positive outcomes for the company regarding performance and relationships at work.

## 1. Introduction

Today, no one doubts that the application of adult attachment to the organizational context has value and sense. Many studies explored adult attachment in relation to organizational behavior and outcomes at the individual, team, and/or organizational levels (Davidovitz et al., 2007; Harms, 2011; Yip et al., 2017; Grady et al., 2019; Lisá and Greškovičová, 2022). Despite the emerging option of transferring adult attachment to the organizational setting, we need to face serious questions and possibly doubts. The most intriguing and interesting question is how we can apply adult attachment to relationships at work. This question covers two basic issues of adult attachment with respect to the work environment. Whether there is conceptual overlap or extension of attachment theory to another context in addition to family and romantic relationships. The second refers to operational limitations of adult attachment at work and the methodological approach to address specific working models at work. In the study, we wanted to provide evidence of the application of adult attachment to the organizational setting, as well as to expand knowledge on how to deal with the interpretation of attachment in the organizational setting.

### 1.1. Attachment models at work

The basic tenant of developing an attachment model based on interactions with significant others remains relevant. However, we can differentiate among three levels in the attachment hierarchy. The ontogenesis of significant relationships and average experience in these relationships are captured in a global or general or dispositional attachment representation (Collins and Read, 1994; Baldwin et al., 1996; Collins et al., 2004). This representation is strongly correlated with overall psychological adjustment (Cozzarelli et al., 2000). The second level of attachment refers to domain-specific attachment representation (such as peer relationships, romantic partners relationships, etc.) that includes the history of specific types of relationship. Lastly, the third level refers to a relationship with a specific person in a relationship-specific model (Overall et al., 2003). To avoid some misunderstanding in the formulation and concepts and in line with Collins and Read (1994) and Baldwin et al. (1996), we use the “attachment style” for the global mental representation and the “attachment orientation” for domain-specific representations of attachment relationships.

Attachment mental representations in the hierarchy are distinct (Overall et al., 2003), yet interconnected, either horizontally or vertically. For example, Klohnen et al. (2005) showed that domain-specific mental representations are connected with global mental representations, and some of them are more than others. In their study, the peer domain contributed more to global mental representation than the parent domain. Relationship-specific models were also shown to shape higher mental representations. This might be called the bottom-up effect, which was also confirmed in a recent study by Dugan et al. (2022).

In the work context, a previous study (Greškovičová et al., 2022) showed that global attachment correlated with domain-specific working model (attachment to colleagues), especially avoidant adult attachment, which was correlated with dismissive attachment to colleagues. Global attachment also predicted attachment to colleagues. Interestingly, a combination of avoidance and anxiety in global adult attachment better predicted preoccupied and dismissive attachment to colleagues.

Moreover, vertical interconnection between domain-specific models is also registered. People securely attached to parents were also securely attached to partners (Klohnen et al., 2005). However, the relationship-specific attachment model does not correlate with other domain-specific attachment models (Sibley and Overall, 2008). Relationship-specific attachment models coming from the same domain are more interconnected than ones from distinct domains (Klohnen et al., 2005; Fraley et al., 2011; Hudson et al., 2015).

We see a difference in how attachment principles are expressed in the work environment compared to other spheres of human life (Mayseless, 2010). In an organizational context, it would be appropriate to analyze specific models to understand the dynamics of attachment in the workplace. The possible examples of work domains are the following: colleagues, leader, workplace. However, there are many studies that use general/global attachment representations or other domain attachment representations to explore organizational outcomes or behavior (Hazan and Shaver, 1990; Richards and Schat, 2011; Mikulincer and Shaver, 2016; Yip et al., 2017). Few researchers helped to solve the issue of applying global or romantic relationship domain models to working settings (Brumbaugh and Fraley, 2007). They brought measurements to explore attachment to the workplace, colleagues, or leaders (Scrima et al., 2014; Molero et al., 2019; Scrima, 2020).

Since prior research has shown the validity and predictive power of specific attachment models (Klohnen et al., 2005; Gruda and Kafetsios, 2020; Lisá et al., 2021), the current study aims to examine specific attachment models in work settings and to specify their functions.

*H1:* We assume that work-specific domains of attachment (colleagues, leader, and workplace) will differ in their function for the workplace and they will differ from romantic domain.

### 1.2. Specific attachment models with work and organizational variables

Given the evidence that there are multiple mental representations of attachment relationships (Baldwin et al., 1996; Brumbaugh and Fraley, 2007), it is also valuable to devote time exploring whether attachment in the workplace is good for predicting or explaining work and organizational behaviors and outcomes.

Specific models in the workplace were under scrutiny by researchers, although to a lesser extent. In terms of domain-specific attachment representation, attachment to colleagues model seems to be related to performance, mental ill-being, and affective experience. For example, in terms of performance, attachment to colleagues is related to instrumental help (Geller and Bamberger, 2009), job performance (Neustadt et al., 2011), engagement (Byrne et al., 2017), organizational commitment (Scrima et al., 2015), and civility (Leiter et al., 2015). In terms of mental well-being and affective experience, attachment is related to trust, hope, and burnout (Simmons et al., 2009; Leiter et al., 2015) and psychological safety (Leiter et al., 2015).

Attachment toward a leader (relationship-specific attachment) is related to followers’ extra and in-role performance (Shanock and Eisenberger, 2006), proactive behavior (Wu and Parker, 2017), organizational citizenship performance (Molero et al., 2013; Lisá et al., 2021). It is also related to job attitudes, such as job satisfaction (Lavy, 2014). Job satisfaction is related to mental health and quality of life (Faragher et al., 2005; Ioannou et al., 2015; Bae, 2021). Regarding mental illness, attachment to the leader is related to followers’ burnout (Lavy, 2014). Furthermore, the transformational leader as an attachment figure also promoted performance at the team level (Lisá and Greškovičová, 2022). However, not only is leadership style important for organizational, group, and follower outcomes, but the personality of leaders is also relevant (Antonakis et al., 2012; Blake et al., 2022).

Another object of attachment in the work setting can be the workplace itself. It is defined as a one-dimensional construct, focusing on the intensity of emotional link and the dynamic interaction between an employee and the organizational environment (Rioux, 2006; Le Roy and Rioux, 2012; Rioux and Pignault, 2013; Scrima et al., 2014, 2017). Workplace attachment refers to specific attachment models (Cozzarelli et al., 2000; Klohnen et al., 2005). Based on the attachment model of Bartholomew and Horowitz (1991), Scrima (2020) defined workplace attachment styles similar to secure, preoccupied, and dismissive. The intensity of workplace attachment is related to adult attachment styles, which provides empirical support for the theoretical assumption that individuals’ bonds with their workplace articulate attachment bonds (Scrima, 2015; Scrima et al., 2017). However, the effects of workplace attachment may be much more directly related to organizational behavior than transferred relationships with other significant people. Previous research shows that workplace attachment is related to organizational citizenship performance, namely affective commitment (Le Roy and Rioux, 2012; Scrima, 2015). This association is corroborated by a positive correlation between organizational citizenship performance and organizational climate (Bahrami et al., 2016; Berberoglu, 2018). Furthermore, workplace attachment is also connected to helping behavior toward colleagues (Le Roy and Rioux, 2012), civic virtue and altruism (Nonnis et al., 2022).

Since prior research has shown the importance of multiple attachment models in organizational settings, we wanted to explore specific adult attachments in the working setting and investigate their associations and predictive power on various organizational variables.

*H2:* We assume that work-specific domains of attachment (colleagues, leader, workplace) will relate to the work attitude (work engagement), work behavior (transformational leadership), and work performance (general performance and citizenship organizational behavior).

### 1.3. Attachment types in the workplace

Lastly, we want to focus on attachment types in the workplace. Based on attachment theory, attachment styles/orientations mirror internal working models (Bartholomew and Horowitz, 1991). There are two fundamental attachment styles, secure and insecure. The insecure attachment style encompasses two or three: preoccupied, dismissive/avoidant, and fearful. The last one is not present in a work setting because it refers to a person with serious mental problems that prevent them from being part of the workforce.

Original work on measuring attachment delineated prototypes as distinct types of attachment (Bartholomew and Horowitz, 1991). Later, some of the tools focused on continuous measurement of dimensions (such as avoidance and anxiety in ECR by Fraley et al., 2000). On the basis of the combinations of these dimension scores, one can calculate the attachment style/orientation. We propose person-centered analysis as a combination of dimensions.

Cluster analysis for attachment seems to be valuable in various settings, such as clinical, counseling, and sport psychology (Pilkauskaite-Valickiene et al., 2011; Alexandris and Tsiotsou, 2012; Einav, 2014). However, in work and organizational setting cluster analysis is not frequent. We believe that person-centered analysis can provide necessary and valuable information on employees and their complex behavior at work.

*H3:* We assume that dimensions of the colleagues domain attachment model will cluster into attachment types at work, according to secure, preoccupied, and avoidant attachment orientations.

## 2. Materials and methods

### 2.1. Type of the study

Based on quantitative approach, we carried out a time-lagged cross-sectional research design. We collected data in two waves with a three-month break. Because all variables were gathered from the same source, the time-lagged design was applied to minimize the risk of common-method variance (Podsakoff et al., 2003).

### 2.2. Participants

The targeted population was adult people with working experience. In the research, convenient and voluntary sampling was used. We published the link to the research on social networks from January until April 2020. According to the smallest subject-to-item ratio for EFA purposes, which is 20:1 (Costello and Osborne, 2005), we aimed to recruit at least 360 participants. They completed online questionnaires and provided online agreement with participation in the research.

One thousand and three hundred and fifty-two participants participated in the research analysis, 35% of men (1% did not share their gender), aged 18–78 years. Participants collaborated with their current leader and team for 6.9 years on average (SD = 7.5), a minimum of 0, and a maximum of 45 years. They worked in various work teams and various work positions.

We divided the battery into participant-friendly batteries into two waves. The first wave included questionnaires AAW, WASQ, SBO, and LSPS. The second wave included questionnaires TFL, COB, GP, UWES, and ECR-R. Participants from the first and the second wave were paired by individual voluntary codes. Subsequently, the participants differed in completing the questionnaires, because of natural drop-out. Some of the participants commented on the ECR-R questionnaire. In the open feedback question, the participants perceived the ECR-R items as too intimate and less acceptable for the working environment. All participants completed the AAW questionnaire (the first in the battery), 844 completed WASQ, 625 SBO, and 261 LSPS. In the second wave, 240 participants completed TFL and COB, 238 GP, 237 UWES, and 235 ECR-R. We refer to measures in the exact order as shown to the participants.

### 2.3. Materials

#### 2.3.1. Attachment measures

The Adult Attachment in the Workplace Questionnaire (AAW; Neustadt and Furnham, 2006; Scrima et al., 2014) was used in its Slovak version and shortened version proposed by Lelkesova and Lisá et al. (2021). It measures three orientations of attachment towards colleagues. The answers are rated on the 5-point Likert scale (1—strongly disagree to 5—strongly agree). The fit indices showed that the data fit the model well (CFI = 0.983; TLI = 0.976; RMSEA = 0.051; SRMR = 0.044).

The Workplace Attachment Styles Questionnaire (WASQ; Scrima, 2020) in the short 9-item form of the Slovak translation (Lisá and Mrázková, 2021) measures three orientations of attachment to the workplace. The model showed the following fit indices CFI = 0.991; TLI = 0.986; RMSEA = 0.043; SRMR = 0.040. Internal consistency was measured by Cronbach’s alpha and McDonald’s omega coefficients for each of the attachment orientations: avoidant *α* = 0.778; *ω* = 0.781; preoccupied *α* = 0.751; *ω* = 0.752; secure *α* = 0.628; *ω* = 0.638. The 5-point Likert scale was used (0—do not agree at all, 4—completely agree).

The Scale of Belonging to the Organization (SBO; Kretová, 2005; Lisá, 2020) consists of six items in two dimensions: belonging to place (*α* = 0.861; *ω* = 0.861) and to people (*α* = 0.798; *ω* = 0.798). The fit indices showed good data fit (*CFI* = 1.000; *TLI* = 1.002; *RMSEA* = 0.000; *SRMR* = 0.020). The Likert scale from 1 (never) to 6 (always) was used.

The Leader as Security Provider Scale (LSPS; Molero et al., 2019) measures the perception of a leader as a secure figure. We used the Slovak translation (Lisá et al., 2021) in a shortened 10-item and 2-factor solution (Mrázková and Lisá, 2022). The CFA showed good data fit indices (CFI = 0.998; TLI = 0.997; RMSEA = 0.028; SRMR = 0.054). Internal consistency was measured using Cronbach’s alpha and McDonald’s omega coefficients for both factors: secure figure (SF) *α* = 0.881; *ω* = 0.887; separation distress (SD) *α* = 0.791; *ω* = 0.797. The 5-point Likert scale was used (0—do not agree at all, 4—completely agree).

Experiences in Close Relationships-Revised Questionnaire (Fraley et al., 2000) in the shortened version of Slovak ECR-R-SK-14 (ECRR; Švecová et al., 2021) measures attachment to romantic partners. The CFA showed acceptable data fit indices; CFI = 0.982; TLI = 0.979; RMSEA = 0.059; SRMR = 0.076. Internal consistency was measured by Cronbach’s alpha and McDonald’s omega coefficients for both attachment styles: anxiety *α* = 0.861; *ω* = 0.862; avoidance *α* = 0.800; *ω* = 0.803. The 7-point Likert scale was used (1—do not agree at all, 7—completely agree).

#### 2.3.2. Organizational variables

The Czech Leadership Questionnaire (TLF; Procházka et al., 2016) measures transformation leadership through 16 items on a 7-point Likert scale, from 1-never to 7-always (Cronbach’s *α* = 0.933; McDonald’s *ω* = 0.934). TFL consists of idealized influence, inspirational motivation, intellectual stimulation, and individual consideration. We used the recommended one-dimensional second-order factor model, calculated as a subscale average (Procházka, 2020). The model fit indices were CFI = 1.000; TLI = 1.001; RMSEA = 0.000; SRMR = 0.056.

The Citizenship Organizational Behavior Questionnaire (COB; Coleman and Borman, 2000) measures citizenship organizational behavior It has three dimensions: interpersonal citizenship performance (COB1) as interpersonal altruism and interpersonal conscientiousness (*α* = 0.828; *ω* = 0.829); Organizational citizenship performance (COB2) as organizational allegiance/loyalty, and organizational compliance (*α* = 0.848; *ω* = 0.855); Job/Task Conscientiousness (COB3) as an extra effort and job dedication (*α* = 0.750; *ω* = 0.755). The data fit showed the acceptable model (CFI = 0.991; TLI = 0.990; RMSEA = 0.038; SRMR = 0.073).

The General Work Performance Questionnaire (GP; Motowidlo and Van Scotter, 1994) measures overall perceived general performance. It has three items on a 7-point Likert scale, with 7 for high performance and 1 for low performance. The mean of the three items formed the overall performance score (*α* = 0.785; *ω* = 0.792). The model does not have enough items for CFA; the fit indices show the perfect fit with the data (CFI and TLI equal 1, RMSEA and SRMR equal 0).

The Utrecht Work Engagement Scale measures work engagement (UWES; Schaufeli and Bakker, 2004). We used a short 9-item questionnaire, with a 7-point Likert scale from 0—never to 6—each day: CFI = 0.999; TLI = 0.998; RMSEA = 0.027; SRMR = 0.055; α = 0.926; ω = 0.927.

### 2.4. Ethical considerations

The survey was anonymous, and the participants were treated according to the ethical standards of the APA and the Declaration of Helsinki. Ethical Committee of the Faculty of Social and Economic Sciences, Comenius University in Bratislava, provided the Statement of Ethical Approval Exemption upon number 823-2/2022. All participants gave their informed consent under the condition of complete anonymity, voluntary provision of information, and the option to leave whenever they wanted without any consequences. Participants agreed to the aggregated data analysis for the research study purpose. The researchers complied with Personal Data Protection Act No. 18/2018, Coll., and internal university regulation Nb. 23/2016. The authors acknowledged compliance with the obligation to ensure informed consent from research participants in a declaration at the end of the article. The researchers are unaware of any foreseeable intended or unintended adverse impact on participants in the study. Participants confirmed their informed consent online. They got information about the length of the questionnaire, and that the data will be analyzed without a possibility to identify individuals and their results.

### 2.5. Data analysis

The alpha level for statistical testing was set to 0.05. To analyze the structure of the measurements, we applied CFA with a 95% confidence interval, 5,000 bootstrap replications, and ULS estimator for ordinal variables (Li, 2016). Data were analyzed in JASP 0.16.3 (RRID:SCR_015823). Then we applied the correlation analysis, independent t-test, exploratory factor analysis, two-step cluster analysis, with log-likelihood as distance measure, with automatically determined number of clusters through IBM SPSS Statistics 22 (RRID:SCR_019096).

## 3. Results

### 3.1. Exploratory factor analysis

The exploratory factor analysis of the variables based on parallel analysis, with Promax rotation (KMO = 0.814), resulted in four factors (Table 1) that explain 56.8% of the variation of the included variables.

**Table 1:** Exploratory factor analysis.

	Work performance	Insecure attachment at work	Perception of leader and secure workplace attachment	Romantic relationship attachment	Uniqueness
COB3	0.913				0.276
COB2	0.880				0.237
COB1	0.795				0.367
GP	0.664				0.610
UWES	0.630				0.333
SOB_Place	0.429	−0.575			0.180
AAW_AV		0.800			0.450
SOB_People		−0.791			0.367
WASQ_PR		0.673			0.488
AAW_PR		0.604			0.485
WASQ_AV		0.598			0.604
AAW_SC		−0.430			0.756
LSPS_SF			0.935		0.243
LSPS_SB			0.834		0.313
TFL			0.645		0.349
WASQ_SC			0.468		0.523
ECRR_ANX				0.725	0.430
ECRR_AV				0.476	0.761
Variation	18.6	18.0	12.2	8.0	56.8

AAW_AV, avoidant attachment dimension; AAW_PR, preoccupied attachment dimension; AAW_SC, secure attachment dimension; WASQ_AV, avoidant workplace attachment dimension; WASQ_PR, preoccupied workplace attachment dimension; WASQ_SC, secure workplace attachment dimension; LSPS_SF, perceiving leader as a secure figure; LSPS_SD, perceiving separation distress from a leader; ECRR_ANX, anxious romantic attachment dimension; ECRR_AV, avoidant romantic attachment dimension; SOB_Place, belonging to place; SOB_People, belonging to people; UWES, work engagement; TFL, transformational leadership; GP, general performance; COB1, interpersonal citizenship performance; COB2, organizational citizenship performance; COB3, job/task conscientiousness. The applied rotation method is Promax.

The dimensions of AAW load one factor that we named “Insecure attachment at work”. The factor is negatively loaded by the scale of belonging to an organization and secure attachment dimensions. It is positively loaded by preoccupied and avoidant attachment dimensions. It expresses 18% of the data variation. The “work performance” factor (18.6%) includes measures of performance and work engagement. The factor “Perception of the leader and secure workplace attachment” includes perceiving the leader as an attachment figure, transformational leadership, and secure workplace attachment. It explains 12.2% of the total variance. The factor “Romantic relationship attachment” includes anxious and avoidant romantic attachment, with 8% of explained variance.

The romantic domain attachment dimensions differ from the work domain attachment dimensions. Attachment to people (colleagues, leader) and workplace attachment do not relate to romantic domain attachment. The results show that the leader domain is related to transformational leadership and perception of a secure workplace. The domain of colleagues is related to the insecure characteristics of the workplace and feelings of belonging. The work performance factor does not include any of the attachment dimensions.

We supported H1.

### 3.2. Correlations

The correlations between the variables, the mean, standard deviations, and reliability coefficients are shown in Table 2. Avoidant attachment at work (AAW) is positively correlated with preoccupied attachment at work (0.457), avoidant workplace attachment (0.391), preoccupied workplace attachment (0.457), distress when separated from a leader (0.126), anxious romantic attachment (0.230) and avoidant romantic attachment (0.152). It negatively correlates with secure attachment at work (−0.303), workplace secure leader attachment (−0.169), secure figure (−0.182), belonging to place (−0.397), belonging to people (−0.551), work engagement (−0.206), transformational leadership (−0.299) and citizenship performance dimensions (−0.324; −0.224; −0.104).

**Table 2:** Correlations, mean, standard deviations, and reliability coefficients.

Variable	1	2	3	4	5	6	7	8	9	10	11	12	13	14	15	16	17	18
1. AAW_AV	—																	
2. AAW_PR	0.457***	—																
3. AAW_SC	−0.303***	−0.276***	—															
4. WASQ_AV	0.391***	0.400***	−0.249***	—														
5. WASQ_PR	0.457***	0.359***	−0.254***	0.527***	—													
6. WASQ_SC	−0.169***	−0.132***	0.248***	−0.063	−0.259***	—												
7. LSPS_SF	−0.182**	−0.134*	0.124	−0.118	−0.228***	0.381***	—											
8. LSPS_SD	0.126*	0.214***	−0.047	0.130	0.049	0.494***	0.567***	—										
9. ECRR_ANX	0.230***	0.404***	−0.271***	0.250***	0.201**	0.150*	−0.058	0.154*	—									
10. ECRR_AV	0.152*	0.225***	−0.199**	0.149*	0.142*	0.017	−0.084	0.101	0.435***	—								
11. SOB_Place	−0.397***	−0.293***	0.367***	−0.282***	−0.419***	0.513***	0.323***	0.210**	0.068	−0.063	—							
12. SOB_People	−0.551***	−0.287***	0.283***	−0.232***	−0.324***	0.299***	0.170*	0.037	−0.024	0.053	0.616***	—						
13. UWES	−0.206**	−0.130	0.201**	−0.168*	−0.429***	0.466***	0.484***	0.319***	0.045	−0.101	0.694***	0.291***	—					
14. TFL	−0.299***	−0.241***	0.171*	−0.199**	−0.272***	0.320***	0.695***	0.358***	−0.159*	−0.191**	0.424***	0.341***	0.520***	—				
15. GP	0.055	−0.146*	0.165*	−0.074	−0.124	0.172*	0.290***	0.222***	−0.104	−0.041	0.227***	0.026	0.350***	0.260***	—			
16. COB1	−0.324***	−0.222***	0.205**	−0.246***	−0.253***	0.230***	0.308***	0.137*	−0.052	−0.174*	0.449***	0.361***	0.508***	0.409***	0.322***	—		
17. COB2	−0.224***	−0.189**	0.201**	−0.215**	−0.331***	0.271***	0.440***	0.206**	−0.046	−0.150*	0.543***	0.267***	0.627***	0.538***	0.428***	0.711***	—	
18. COB3	−0.104	−0.112	0.153*	−0.149*	−0.273***	0.319***	0.396***	0.297***	0.060	−0.030	0.528***	0.177**	0.633***	0.413***	0.489***	0.605***	0.696***	
*M*	2.44	2.23	3.49	1.21	1.35	1.95	2.43	1.34	2.69	5.43	3.46	3.35	3.68	4.81	5.36	3.60	3.66	3.38
SD	0.75	0.73	0.67	0.91	0.89	0.82	0.73	0.90	1.24	1.01	0.95	0.97	1.14	1.09	0.85	0.65	0.61	0.65
Cronbach’s *α*	0.78	0.66	0.56	0.78	0.75	0.63	0.88	0.79	0.86	0.80	0.86	0.80	0.93	0.93	0.76	0.83	0.85	0.75
McDonald’s *ω*	0.78	0.66	0.57	0.78	0.75	0.64	0.87	0.80	0.86	0.80	0.86	0.80	0.93	0.93	0.79	0.83	0.86	0.76

AAW_AV, avoidant attachment dimension; AAW_PR, preoccupied attachment dimension; AAW_SC, secure attachment dimension; WASQ_AV, avoidant workplace attachment dimension; WASQ_PR, preoccupied workplace attachment dimension; WASQ_SC, secure workplace attachment dimension; LSPS_SF, perceiving the leader as a secure figure; LSPS_SD, perceiving separation distress from a leader; ECRR_ANX, anxious romantic attachment dimension; ECRR_AV, avoidant romantic attachment dimension; SOB_Place, belonging to place; SOB_People, belonging to people; UWES, work engagement; TFL, transformational leadership; GP, general performance; COB1, interpersonal citizenship performance; COB2, organizational citizenship performance; COB3, Job/Task Conscientiousness; M, mean; SD, standard deviation. *** *p* < 0.001; ** *p* < 0.01; * *p* < 0.05.

Preoccupied attachment at work (AAW) is positively correlated with avoidant and preoccupied workplace attachment (0.400; 0.359), distress when separated from a leader (0.214), anxious and avoidant romantic attachment (0.404; 0.225). It negatively correlates with secure attachment at work (−0.276), secure workplace attachment (−0.132), secure leader figure (−0.134), belonging to place and people (−0.293; −0.287), work engagement (−0.130), transformational leadership (−0.241), general performance (−0.146), interpersonal and organizational citizenship performance (−0.222; −0.189).

Secure attachment at work positively correlates with secure workplace attachment (0.248), belonging to place and people (0.367; 0.283), work engagement (0.201), transformational leadership (0.171), general performance (0.165), and citizenship performance dimensions (0.205; 0.201; 0.153). It negatively correlates with avoidant and preoccupied workplace attachment (−0.249; −0.254), anxious and avoidant romantic attachment (−0.271; −0.199).

We supported H2.

### 3.3. Cluster analysis

**Figure 1:**
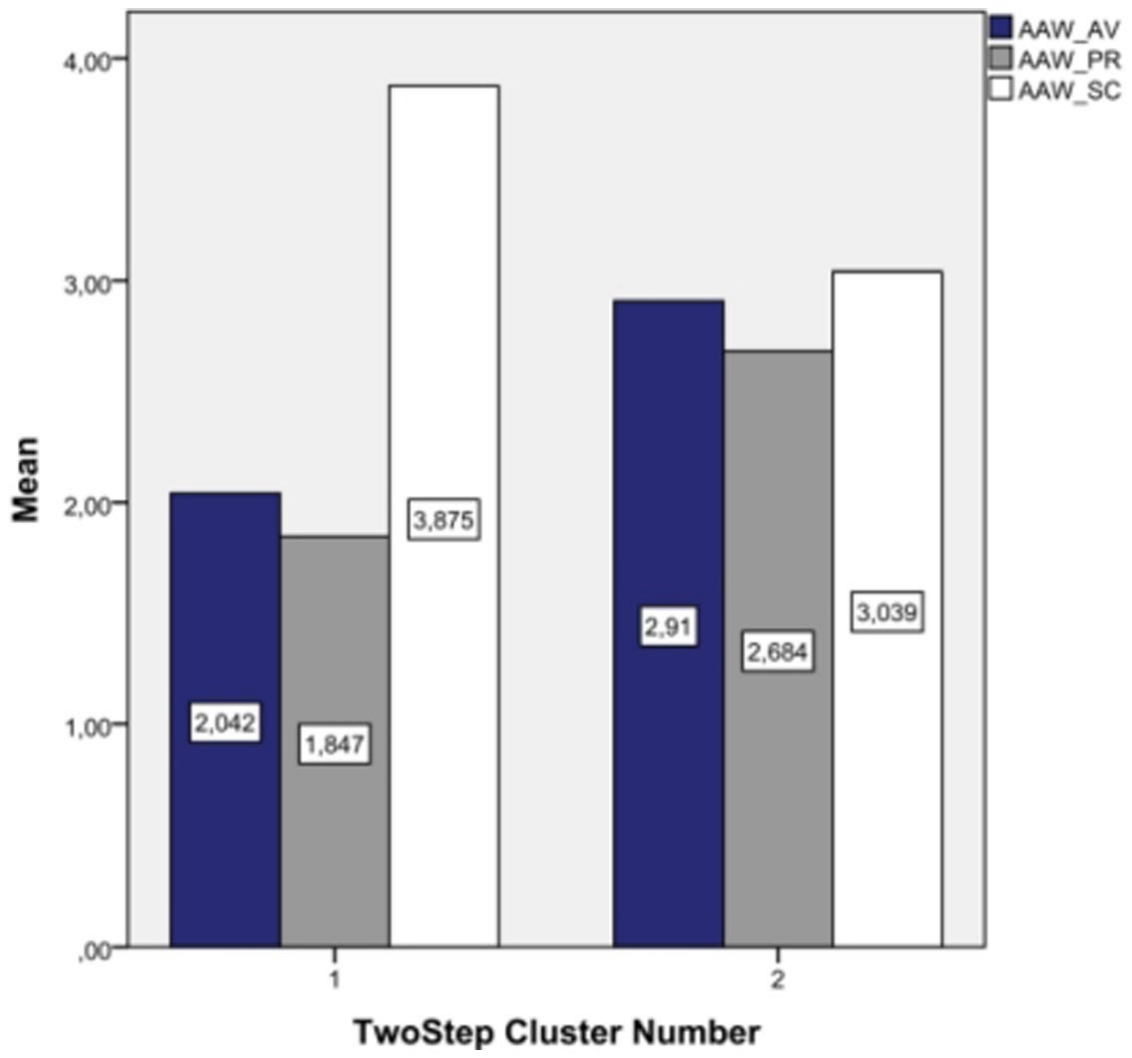
Means of AAW dimensions in clusters.

We input three variables, AAW attachment orientations, into the cluster analysis. The results showed two clusters. 736 people (54.5%) belonged to the first cluster and 615 (45.5%) to the second. Ratio of sizes (largest cluster to smallest cluster) = 1.2. Cluster quality (the silhouette measure of cohesion and separation) is fair (average silhouette = 0.5). The input (predictor) importance of the dimensions for the results was: secure dimension 100%, avoidant dimension 81%; preoccupied dimension 80%. Figure 1 represents the means of the AAW dimensions in clusters of attachment to colleagues at work.

In Cluster 1 participants have high secure orientation and low preoccupied and avoidant orientations. We named this cluster “securely attached type” Compared to Cluster 1, employees in Cluster 2 have similar levels of all three attachment orientations. Based on the following results (Table 3), we named the Cluster as “insecurely attached type”.

**Table 3:** Differences between types.

	Type	*M*	SD	SE	*t*	*df*	*p*	Cohen’s *d*
AAW_AV	Type 1	2.042	0.591	0.022	−25.991	1,349	< 0.001	−1.420
	Type 2	2.910	0.634	0.026				
AAW_PR	Type 1	1.847	0.523	0.019	−25.841	1,349	< 0.001	−1.412
	Type 2	2.684	0.667	0.027				
AAW_SC	Type 1	3.875	0.498	0.018	29.611	1,349	< 0.001	1.618
	Type 2	3.039	0.539	0.022				
WASQ_AV	Type 1	0.855	0.791	0.039	−12.259	841	< 0.001	−0.844
	Type 2	1.560	0.877	0.043				
WASQ_PR	Type 1	1.012	0.808	0.039	−11.689	841	< 0.001	−0.805
	Type 2	1.677	0.844	0.041				
WASQ_SC	Type 1	2.127	0.837	0.041	6.495	841	< 0.001	0.447
	Type 2	1.770	0.759	0.037				
LSPS_SF	Type 1	2.511	0.720	0.065	1.663	258	0.098	0.206
	Type 2	2.360	0.740	0.063				
LSPS_SD	Type 1	1.185	0.845	0.076	−2.566	258	0.011	−0.319
	Type 2	1.468	0.924	0.079				
ECRR_ANX	Type 1	2.259	1.127	0.109	−5.103	233	< 0.001	−0.668
	Type 2	3.045	1.216	0.107				
ECRR_AV	Type 1	2.350	1.038	0.100	−3.044	233	0.003	−0.399
	Type 2	2.746	0.952	0.084				
SBO_Place	Type 1	3.855	0.880	0.053	10.095	622	< 0.001	0.813
	Type 2	3.136	0.888	0.048				
SBO_People	Type 1	3.771	0.895	0.054	10.818	622	< 0.001	0.871
	Type 2	3.000	0.876	0.047				
UWES	Type 1	3.877	1.140	0.110	2.513	234	0.013	0.328
	Type 2	3.508	1.109	0.098				
TFL	Type 1	5.024	1.144	0.110	2.794	237	0.006	0.363
	Type 2	4.633	1.019	0.089				
GP	Type 1	5.503	0.745	0.072	2.441	235	0.015	0.318
	Type 2	5.235	0.914	0.080				
COB1	Type 1	3.797	0.586	0.056	4.402	237	< 0.001	0.572
	Type 2	3.436	0.667	0.058				
COB2	Type 1	3.797	0.565	0.054	3.228	236	0.001	0.420
	Type 2	3.547	0.621	0.055				
COB3	Type 1	3.492	0.641	0.061	2.404	237	0.017	0.312
	Type 2	3.292	0.640	0.056				

AAW_AV, avoidant attachment dimension; AAW_PR, preoccupied attachment dimension; AAW_SC, secure attachment dimension; WASQ_AV, avoidant workplace attachment dimension; WASQ_PR, preoccupied workplace attachment dimension; WASQ_SC, secure workplace attachment dimension; LSPS_SF, perceiving leader as a secure figure; LSPS_SD, perceiving separation distress from a leader; ECRR_ANX, anxious romantic attachment dimension; ECRR_AV, avoidant romantic attachment dimension; SOB_Place, belonging to place; SOB_People, belonging to people; UWES, work engagement; TFL, transformational leadership; GP, general performance; COB1, interpersonal citizenship performance; COB2, organizational citizenship performance; COB3, Job/Task conscientiousness.

Types differ in all measured variables, except perceiving the leader as a secure figure (Table 3). Cluster 1, securely attached type, has all insecure attachment dimensions low and secure attachment dimension high. The directions of differences are the same in dimensions of workplace attachment and romantic attachment orientations. The securely attached employee expresses higher work engagement, transformation leadership, general, and citizenship performance than the insecurely attached employee. Participants in this cluster also have lower separation distress. Lastly, the perception of the leader as a secure figure is not related to attachment orientations at work. Attachment to colleagues is not related to the perception of the leader as a secure attachment figure.

Based on the large effect size in differences, we can characterize the securely attached type compared to the insecurely attached type as the type with higher levels of secure attachment to colleagues, greater belonging to the organization and higher interpersonal citizenship performance. On the other hand, it has a lower avoidant and preoccupied attachment to colleagues, a lower avoidant and preoccupied workplace attachment, and a lower anxious romantic orientation than the insecure type.

Although we did not support the three clusters (H3) prediction, we showed that working people clustered into two distinct classes.

## 4. Discussion

Work-specific attachment models differ from the romantic-domain attachment model. Moreover, the work-specific attachment models differ among themselves. Therefore, it is necessary to explore specific attachment models in the workplace that relate directly to work, such as colleagues, leaders, and workplaces. The romantic domain attachment model seems to be inappropriate for examination in an organizational setting because it is not associated with any work-specific attachment models.

Relationships at work operate differently from relationships in private life. The working model of the romantic relationship domain does not function in an organizational context. It may even be inadequate to use it in the work environment to capture attachment in the work setting. This was statistically confirmed by different EFA factors. Furthermore, participants indicated that they did not want to complete the romantic attachment questionnaire as they perceived it inappropriate in a workplace.

Domain attachments are distinct, and they seem to be separate mental representations. Therefore, they should be considered and measured separately. We cannot replace one domain attachment orientation with another. Consequently, it is essential to use methods designed for a given domain attachment.

Not only did different domains (domains of colleagues and romantic partners) prove conceptually and empirically distinct. Even the relationship-specific attachment to the leader and domain-specific attachment to colleagues are distinct. Research suggests that specific models may be interconnected (Fraley et al., 2011; Hudson et al., 2015; Dugan et al., 2022). Thus, we aimed to answer whether attachment towards colleagues and toward a leader fell into the same domain of mental representations. According to our results, they are distinct. Colleagues and leaders form two separate domains in the organizational context that should be treated as such. Additionally, attachment to a leader converges with leadership style and secure workplace attachment. It suggests that a leader creates a sense of secure workplace. On the other hand, attachment to colleagues might be more important in insecure workplace attachment and insecure belonging.

Based on attachment to colleagues, it is possible to distinguish attachment orientations among working adults. The securely attached ones have the secure orientation twice as high as the two insecure orientations. The insecurely attached types have all three attachment dimensions at the same level. The results are consistent with the interpretation that the distinction between avoidant and preoccupied insecure orientations at work is very small and nonsignificant (Neustadt et al., 2011). However, it contradicts the theoretical background (Mikulincer and Shaver, 2016) and empirical evidence (Littman-Ovadia et al., 2013; Wardecker et al., 2016; Read et al., 2018) that suggests that there is more than one insecure style or orientation. We did not find solid evidence of differentiating between avoidant and preoccupied attachment orientations in insecure orientation (Neustadt and Furnham, 2006; Neustadt et al., 2011), despite the good fit indices of the three-factor model (Scrima et al., 2014).

Interpretation of the AAW orientations/dimensions cannot simply be interpreted based on the level of one dimension. All three dimensions must be considered, and their combination is valuable for interpreting and understanding the behavior of an employee. The combination of dimensions shows that the securely attached employee empirically mirrors the theoretical conception of a securely attached individual. They seem to profit from their secure attachments at work in all the variables studied, ranging from various relationships with colleagues, romantic partners, belonging to people and places ending with different performance. They have much better relationships at work with colleagues and toward the workplace, and they also show better romantic relationships and interpersonal citizenship performance than insecurely attached individuals. Securely attached types in the workplace showed greater work engagement, transformation leadership, general, and especially citizenship performance. Citizenship performance with large effect size differed in two types. It seems to be a typical kind of performance when we speak about the work-domain attachment model.

The insecurely attached type suggests that there may be some variability in using the attachment orientations. Attachment styles were previously taken as trait characteristics. A common notion was spread that there was one single style prevalent in a given relationship or relationships. However, with more and more evidence that attachment mental representations are hierarchical (e.g., Baldwin et al., 1996; Brumbaugh and Fraley, 2007; Sibley and Overall, 2008) and context dependent, it seems that people are not so strict in using only one preferential style in attachment relationships. As Baldwin et al. (1996) found, almost half of the participants reported using three attachment styles (secure, preoccupied, dismissive), and the majority (88%) of the participants reported using two attachment styles in one attachment relationship. In this study, approximately half of the participants have a prevalent attachment orientation (that is, secure). The insecurely attached type reported the same level of three attachment orientations (secure, preoccupied, and avoidant) in relationships with colleagues. We can conclude that the variability in attachment style is not only vertical (depending on the hierarchy level), but horizontal as well. People might operate with different orientations in a relationship (not only one single one) depending on the context.

### 4.1. Theoretical, practical, and research implications

This study advances our understanding of attachment working models and their application in the organizational context. The results contribute to hierarchical attachment mental presentations and show the distinction in attachment working models at work. We respond to the call for a greater clarification of specific attachment working models (Dugan et al., 2022) by recognising their value in practise and research of organizations.

The study also illustrates the application of the attachment perspective in the organizational context and the importance of choosing a reasonable approach to study attachment in the workplace. It is valuable to distinguish between insecurely and securely attached to co-workers. We suggest not to rely on eye-ball inspection of received points in dimensions of attachment orientation, but on the profile of all dimensions together.

Furthermore, this study suggests fostering secure attachment to colleagues, since it brings together more positive outcomes for the company in terms of performance and relationships at work. A leader is the figure that represents the security of the workplace. On the other hand, colleagues play a role in sharing worries and anxiety. The twofold higher dimension of security attachment compared to insecure attachment dimension means better performance, work engagement, and perceived transformational leadership in employees.

This study also has some limitations. Convenience sampling, cross-sectional collection, and self-reported instruments might result in distortion of the sample and the data collected.

In future studies, more attachment objects in the workplace may offer a more comprehensive view of organizational variables and attachment models. For example, Grady et al. (2019) indicate that relational attachment is the best predictor of performance. But they do not differentiate between the attachment to colleagues and the leader. The theory talks about various objects, but empirically we have confirmed a few of them. However, there are some new tips on attachment objects, for example, attachment to robots (You and Robert, 2018; Law et al., 2022). This is a very new area of attachment research.

Future research could also examine the empirical relationships among global, domain and specific attachment models, since the “paradigm” of the attachment style being the only one is shattered and more and more scholars are inclined to accept the malleability of attachment orientations (Baldwin et al., 1996; Cozzarelli et al., 2000; Rice et al., 2020).

Future research could also focus on the role of attachment in the workplace in mental health and mental health problems. As stated previously, there are some studies that showed a connection of attachment to mental ill-being characteristics (Simmons et al., 2009; Towler and Stuhlmacher, 2013; Kafetsios et al., 2014; Leiter et al., 2015; Vîrgă et al., 2019) or even adult trauma (Maté and Maté, 2022).

## Data availability statement

The data analyzed in this study is subject to the following licenses/restrictions: the data sets generated and analysed during the current study are available from the corresponding author upon reasonable request. Requests to access these datasets should be directed to KG, katarina.greskovicova@fses.uniba.sk.

## Ethics statement

Ethical review and approval was not required for the study on human participants in accordance with the local legislation and institutional requirements. The patients/participants provided their written informed consent to participate in this study.

## Author contributions

KG: conceptualization, development of design and methodology, writing—original draft, data curation, and writing—review and editing. EL: conceptualization, development of design and methodology, data curation, data analysis, and writing—review and editing. All authors contributed to the article and approved the submitted version.

## Conflict of interest

The authors declare that the research was conducted in the absence of any commercial or financial relationships that could be construed as a potential conflict of interest.

## Publisher’s note

All claims expressed in this article are solely those of the authors and do not necessarily represent those of their affiliated organizations, or those of the publisher, the editors and the reviewers. Any product that may be evaluated in this article, or claim that may be made by its manufacturer, is not guaranteed or endorsed by the publisher.
